# Particle size effects in the thermal conductivity enhancement of copper-based nanofluids

**DOI:** 10.1186/1556-276X-6-217

**Published:** 2011-03-14

**Authors:** Michael Saterlie, Huseyin Sahin, Barkan Kavlicoglu, Yanming Liu, Olivia Graeve

**Affiliations:** 1 Kazuo Inamori School of Engineering, Alfred University, 2 Pine Street, Alfred, NY 14802, USA; 2 Advanced Materials and Devices, Inc., 4750 Longley Lane #104, Reno, NV 89502, USA

## Abstract

We present an analysis of the dispersion characteristics and thermal conductivity performance of copper-based nanofluids. The copper nanoparticles were prepared using a chemical reduction methodology in the presence of a stabilizing surfactant, oleic acid or cetyl trimethylammonium bromide (CTAB). Nanofluids were prepared using water as the base fluid with copper nanoparticle concentrations of 0.55 and 1.0 vol.%. A dispersing agent, sodium dodecylbenzene sulfonate (SDBS), and subsequent ultrasonication was used to ensure homogenous dispersion of the copper nanopowders in water. Particle size distribution of the copper nanoparticles in the base fluid was determined by dynamic light scattering. We found that the 0.55 vol.% Cu nanofluids exhibited excellent dispersion in the presence of SDBS. In addition, a dynamic thermal conductivity setup was developed and used to measure the thermal conductivity performance of the nanofluids. The 0.55 vol.% Cu nanofluids exhibited a thermal conductivity enhancement of approximately 22%. In the case of the nanofluids prepared from the powders synthesized in the presence of CTAB, the enhancement was approximately 48% over the base fluid for the 1.0 vol.% Cu nanofluids, which is higher than the enhancement values found in the literature. These results can be directly related to the particle/agglomerate size of the copper nanoparticles in water, as determined from dynamic light scattering.

## Background

It is well-known that the thermal conductivity of fluids, such as water or ethylene glycol, can be enhanced through the addition of nanoparticles. These two-component systems have effective thermal conductivities that include the thermal conductivity of the pure materials, the composition of the mixture, and the shape of the dispersed particles, as follows [1]:(1)

in which *K*, *K*_1_, and *K*_2 _are the thermal conductivities of the nanofluid, the dispersed phase, and the base fluid, respectively, *n *is an empirical shape factor dependent on the sphericity of the particles, and *V*_2 _is the volume fraction of the particles. This model includes the effect of particle shape and size and has been applied to a copper/water nanofluid system with three different sphericity factors, showing that the thermal conductivity of a nanofluid depends on both the particle volume fraction and the shape [2].

A comparison of particle shape in SiC nanofluids of water and ethylene glycol has been completed with powders of spherical morphology of 26-nm average crystallite size and cylindrical morphology of 600-nm average crystallite size along the fiber axis direction [3]. It was concluded that because heat transfer occurs between the surface of the particles and the fluid, heat transfer is more efficient for a system with a larger interfacial area. Therefore, smaller crystallites will exhibit the greatest amount of thermal conductivity enhancement. However, this conclusion does not take into consideration the effect of agglomeration of the powders in the fluid. If the powders are heavily agglomerated, then heat transfer is possibly hindered, since transfer will only occur on the surfaces of the agglomerates, even if the powder crystallite sizes are in the nanometer range.

In nanofluids of Al_2_O_3_/water, an increase of 12% in thermal conductivity enhancement was observed with the addition of Al_2_O_3 _powders with crystallites of 28-nm average size at a particle volume fraction of 3% [4]. Other studies of this same system, with powders of 13 and 38 nm Al_2_O_3 _average crystallite size exhibited a 20% and 8% thermal conductivity enhancement [5,6]. The effect can be attributed to the phonon mean free path in the nanofluid [7] such that in a nanofluid containing nanoparticles of a crystallite size much different from the phonon mean free path, the thermal conductivity will increase with decreasing crystallite size, while in a nanofluid containing nanoparticles less than or equal to the mean free path, the thermal conductivity will be reduced with crystallite size reduction due to the scattering of phonons. None of these studies reported the level of agglomeration (i.e., the size of the particle/agglomerates) in the nanofluid. Since powders tend to heavily agglomerate in polar fluids, a comparison connected to the agglomeration of the particles and not just to crystallite size, cannot be ascertained without proper particle/agglomerate size distribution analysis [8-14].

Table 1 summarizes results on crystallite and particle sizes, if reported, of several copper-based nanofluids. Of the studies that incorporated particle size distribution analysis, only Sinha *et al. *[8] and Wang *et al. *[15] provided a complete description of instrumental parameters for analysis of the particle size distribution in the fluids. The Cu/and Fe/ethylene glycol nanofluids from Sinha *et al. *[8] exhibited particle sizes of 400-600 nm. In the investigation by Wang *et al. *[15], the particle size average for the fluids prepared without sodium dodecylbenzene sulfonate (SDBS) as dispersant was greater than 1 μm, while the fluids with SDBS exhibited a particle size average around 200 nm.

**Table 1:** Crystallite and particle size results in copper-based nanofluids

Material	Dispersant	Crystallite size (nm)	Particle size (nm)	Reference
Cu prepared using oleic acid	SDBS	15-45	120-200	This study
Cu prepared using CTAB	SDBS	10-37	60-100	This study
Cu	None	30-40	400-500	Sinha *et al. *[8]
Cu	None, SDBS	25	~5,500; ~150	Wang *et al. *[15]
Cu	None	50-100	Not reported	Liu *et al. *[16]
Cu	None	10	Not reported	Eastman *et al. *[17]
Cu	None	100	Not reported	Xuan and Li [18]
Cu	None	25-30	Not reported	Velasco *et al. *[19]
Cu	None, SDBS	25-60	5,560; 130	Li *et al. *[20]
Cu	PVP	5-10	Not reported	Yu *et al. *[21]

In this study, we prepared copper nanopowders and then incorporated these powders into water, with SDBS as a dispersant, for the formation of well-dispersed nanofluids. The copper nanoparticles were produced through the reduction of copper (II) chloride with sodium borohydride in the presence of a surfactant [i.e., oleic acid or cetyl trimethylammonium bromide (CTAB)]. After preparation of powders using various surfactant concentrations, the optimal samples were chosen based on phase and particle size criteria. The small agglomerate sizes for the 0.55 and 1.0 vol.% copper nanofluids exhibited thermal conductivity enhancements of up to 48% over the base fluid with a mean thermal conductivity of 0.89 W/m·K, higher than the enhancement values found in other studies, as will be discussed later. We conclude that minimization of agglomerate size in nanofluids is important in order to take full advantage of the presence of nanopowders in the base fluid.

The main experimental result and contribution of this work to the nanofluid field is that excessive aggregation of the dispersed phase reduces the effectiveness of the produced nanofluid through the settling of particles and disruption of nanofluid flow during thermal conductivity testing. While this has been hypothesized extensively, our work shows definite experimental results to support this. When testing the thermal conductivity of the nanofluids, the increase in particle loading for the oleic acid powders from 0.55 to 1.0 vol.%, results in rapid settling due to increased agglomeration, as carefully determined by dynamic light scattering measurements.

## Experimental methodology

The copper nanoparticles were synthesized using a chemical precipitation technique using a custom benchtop reactor system. Two separate aqueous solutions containing the precursors were prepared and subsequently mixed inside the reactor. A mass of 16.10 g of copper (II) chloride (99+%, Alfa Aesar, Ward Hill, MA, USA) was added to 80 mL of de-ionized water under constant stirring. After 30 min of stirring, a surfactant was added and allowed to mix into the aqueous metal salt solution. Two different surfactants were tested. Oleic acid (MP Biomedical LLC, Solon, OH, USA) and CTAB (high purity grade, AMRESCO Inc., Solon, OH, USA) exhibit very different capping characteristics and were chosen because of their successful stabilization of metal nanoparticles in a variety of studies [18,22-31]. Various amounts of the two surfactants were added to the aqueous copper salt solution to observe the effect of surfactant concentration on particle size and oxidation protection in the final nanofluids. For the optimized nanofluids tested for thermal conductivity, the amounts were 1.67 g of oleic acid and 0.57 g of CTAB. Separately, 11.86 g of sodium borohydride (98%, Alfa Aesar, Ward Hill, MA, USA) were dissolved in 220 mL of de-ionized water. To prevent hydrolysis of the reductant solution, the pH of the borohydride solution was maintained at 11-12 through the addition of 2 M sodium hydroxide (98%, Alfa Aesar, Ward Hill, MA, USA). The reductant solution was then added into the reactor under vigorous stirring with nitrogen purging for 5-10 min. The aqueous salt solution was subsequently added under constant stirring. A vigorous reaction, with temperatures in excess of 75°C, was observed as the copper ions were reduced to metal under the following reaction:

Due to the rapid release of hydrogen during the reaction and the presence of bubbling nitrogen gas, significant foam was formed upon reduction of the copper salt/surfactant solution. To counteract this foaming, octyl aldehyde (98%, Sigma Aldrich, St. Louis, MO, USA), a known de-foaming agent, was added as needed throughout the process.

Once the reaction was complete, the fluid was removed from the reactor, emptied into 50-mL centrifuge tubes, and centrifuged using an Eppendorf Centrifuge 5810 (Eppendorf, Hamburg, Germany 22339) for 10 min at 11,000 rpm. The clear supernatant liquid was discarded and a solution of 50/50 semiconductor grade methanol (99.9%, Alfa Aesar, Ward Hill, MA 01835, USA) and de-ionized water was added to the centrifuge tubes in order to remove sodium chloride and the surfactant from the surfaces of the particles. These tubes were shaken vigorously for 5 min. A second methanol/water wash and a final methanol wash were applied on the powders while decanting the supernatant after each centrifugation. The copper powders were then placed in a vacuum dessicator for 2 to 3 days. Once dry, the powders were ground by hand using a mortar and pestle.

In later experiments, the batch sizes were increased. This required one solution to contain 67.08 g of CuCl_2 _in 200 mL of de-ionized water and a separate solution of 148.86 g of sodium borohydride in 300 mL of de-ionized water. The same molar ratios for the surfactants were used for the production of the larger batch sizes, resulting in amounts of 6.94 g of oleic acid and 1.29 g of CTAB.

Two different nanofluids, of 0.55 and 1.0 vol.% copper concentrations in water, were prepared. For preparation of the 0.55 vol.% copper nanofluid with 15 wt.% dispersant, 2.61 g of SDBS (Sigma Aldrich, St. Louis, MO, USA) was added to 296 mL of de-ionized water and allowed to stir at a slow speed, so that a high shearing force did not result in bubbles on the surface. A mass of 14.78 g of copper powder was then slowly added to the dispersant solution under slow stirring and allowed to mix for 1 h. The 1.0 vol.% copper nanofluid contained 4.74 g of SDBS and 26.88 g of copper powder in 292 mL of de-ionized water. The fluid was transferred to a jacketed reaction vessel and then ultrasonicated using an Ultrasonic Processor (Ace Glass, Vineland, NJ, USA) for 50 min with amplitude of 70%, pulsed on for 3 s, then off for 3 s.

Particle size measurements were performed on a Microtrac Nanotrac Ultra dynamic light scattering system (Microtrac Inc., Montgomeryville, PA, USA). The Microtrac Nanotrac Ultra instrument measures the particle size distribution in solution, with measurement capability from 0.8 nm to 6.5 μm. Multiple measurements were done on each sample, using the appropriate parameters determined by the estimated particle size range and fluid viscosity. With these parameters, the measurements were taken at a run time of 30 s. At least five measurements were taken for each sample and then averaged to produce accurate particle size distribution analysis for each sample as is recommended by the instrument manufacturer and in conjunction with ASTM standard E2490-09.

**Figure 1:**
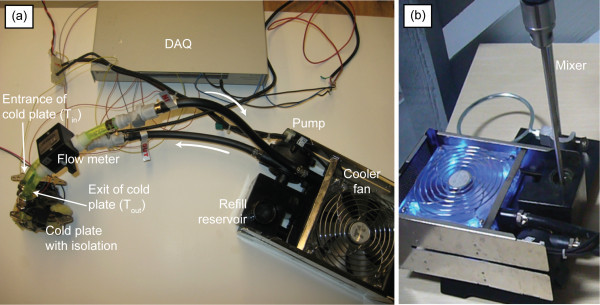
**Experimental setup for thermal conductivity characterization of the nanofluids**.

A commercially available liquid computer cooling system [32] was retrofitted with instrumentation in order to evaluate the thermal performance of the nanofluid, as shown in Figure 1(a). This cooling unit consists of a pump, radiator, cooler fan, refill reservoir, and cold plate. Validation and calibration of the instrument was completed by testing the thermal conductivity of de-ionized water and comparing the output with expected thermal conductivity data. In order to eliminate settling of the nanoparticles in the reservoir, a mixer, illustrated in Figure 1(b), was added to the experimental setup. Up to 200,000 cycles were continuously tested using our dynamic experimental setup. Experimental results show that the change in thermal conductivity of the nanofluid over the entire test (i.e., 200,000 cycles) is less than 1%, thus, our system is highly accurate.

## Results and discussion

**Figure 2:**
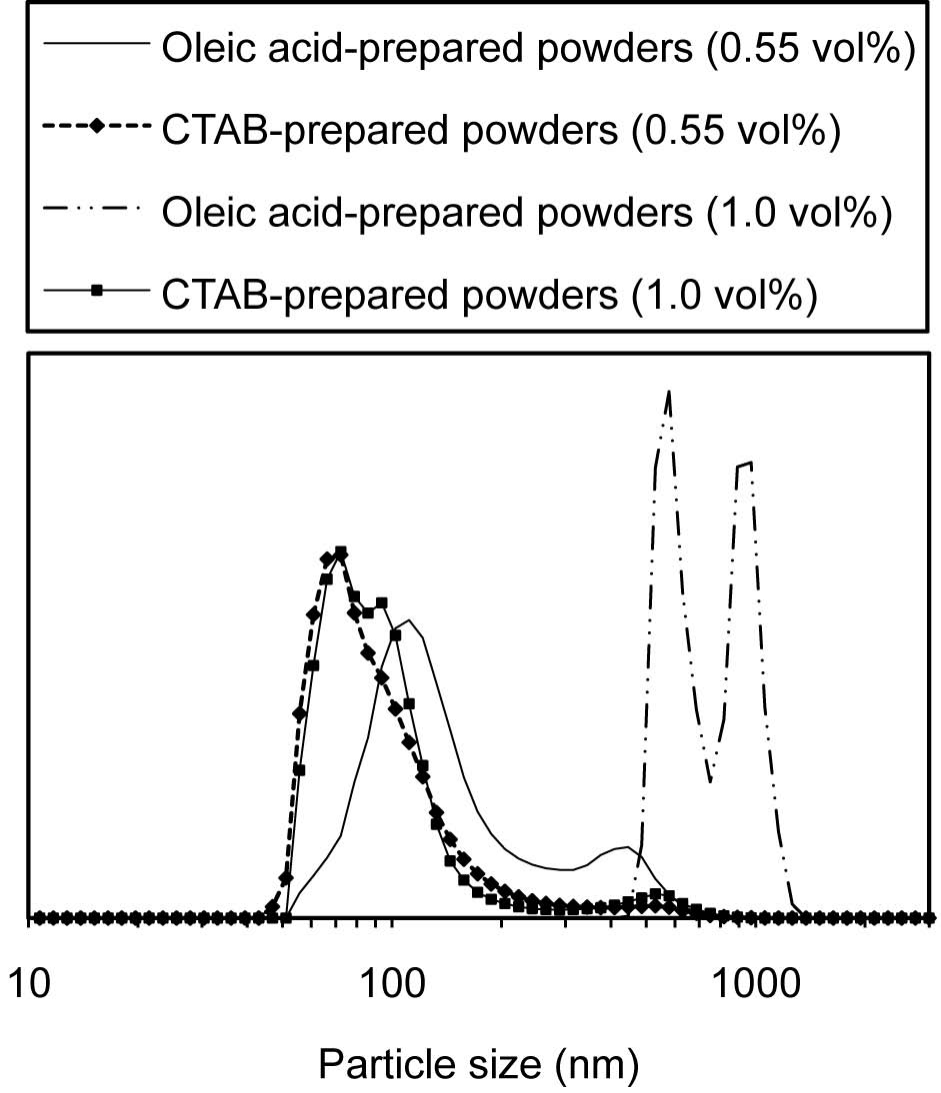
**Particle size distribution**. Particle size distribution measurements for the nanofluids manufactured using oleic acid- and CTAB-prepared copper powders dispersed in water and 15 wt.% SDBS.

Particle size distributions for the 0.55 and 1 vol.% copper nanofluids are displayed in Figure 2. The formation of such small agglomerates, ~120 and ~80 nm on average for the oleic acid- and CTAB-prepared powders, respectively, at a particle loading of 0.55 vol.%, is ideal for the production of effective nanofluids that can exhibit excellent dispersion stability. Smaller agglomerates will stay dispersed within the fluid for a much longer period of time over that of larger, micrometer-sized agglomerates. When increasing the nanoparticle loading to 1.0 vol.%, the nanofluids of oleic acid-prepared powders become heavily agglomerated, increasing the particle size from 120 to 800 nm. This increase in size causes settling of the agglomerates in the nanofluid, which results in clogging during the thermal conductivity measurements. The reasons for the agglomeration of the oleic acid-prepared powders in water will be discussed in a separate report and is generally connected to the surface characteristics of the nanopowders. On the other hand, the nanofluids of CTAB-prepared powders are only slightly more agglomerated at the larger particle loading, with a particle size average of 107 nm. It should be mentioned that the crystallite sizes of the powders in this study are similar to the studies presented in Table 1 and ranges from 10 to 50 nm. Thus, the resultant thermal conductivity differences we have obtained are due to differences in the particle/agglomerate size of the powders in the nanofluid and not to differences in the crystallite size which is, in any case, an approximate average of the primary particle size and most certainly has a distribution. A full analysis and discussion of crystallite size in our powders will be presented in a separate report.

There are several accepted measurement methodologies for thermal conductivity of a nanofluid. In this study, a flow cell experimental setup (Figure 1) was used for the evaluation of dynamic thermal conductivity of the nanofluids. This experimental setup allows for the continuous flow of the nanofluid between an insulated heating element and a cooling fan. Two thermoelectric modules were affixed to both walls of the heating channel and connected to a power supply. The power supply created an electric current which increased the temperature of the walls in the heating channel. A dc pump supplied the circulation of the nanofluid within the closed loop under laminar flow conditions, Re < 2,300. When the nanofluid was passed through the heating channel, it absorbed some of the heat, increasing the temperature of the nanofluid, and then was subsequently cooled back to room temperature by the cooling fan. The temperature of the nanofluid and the walls were monitored by four thermocouples located at the walls and at the inlet and outlet channels of the heating channel. By comparing the steady-state channel wall temperature, the effectiveness of a nanofluid was evaluated.

The pipe surface of the apparatus is hydraulically smooth, therefore, the thermal conductivity of the fluid flowing nearest the wall can be expressed as:(2)

where, *h *is the heat transfer coefficient, *D*_H _is the hydraulic diameter, and Nu is the Nusselt number. The hydraulic diameter is most commonly used in systems with flow in noncircular tubes or channels, and is proportional to the cross-sectional area of the pipe divided by the perimeter of the cross section. The heat transfer coefficient, which is used to calculate the heat transfer, is expressed as:(3)

where, Δ*Q *is the heat input, *A *is the heat transfer surface area, and Δ*T *is the temperature difference between the solid surface and the surrounding fluid. The Nusselt number (Nu) is the ratio of convective to conductive heat transfer across the wall within the channel and is directly proportional to the convective heat transfer coefficient and the hydraulic diameter of the tube, while indirectly proportional to the thermal conductivity of the fluid. Calibration of the experimental setup was validated by testing the system with pure de-ionized water using a Nu of 5.9 for the calculations. A Nusselt number of 5.9 provides a reasonable estimate of *k *for pure water, but it may not be as accurate for nanofluids [33]. Thus, there is likely some error in the determination of the nanofluid thermal conductivities. The thermal conductivity values of de-ionized water for three independent measurements were 0.61, 0.58, and 0.63 W/m·K, with errors of 1.67%, -3.33%, and 5.00%, respectively. For the case of the nanofluid thermal conductivities, the experimental errors are likely slightly greater than 5%, although the exact magnitude of these errors is not known.

**Figure 3:**
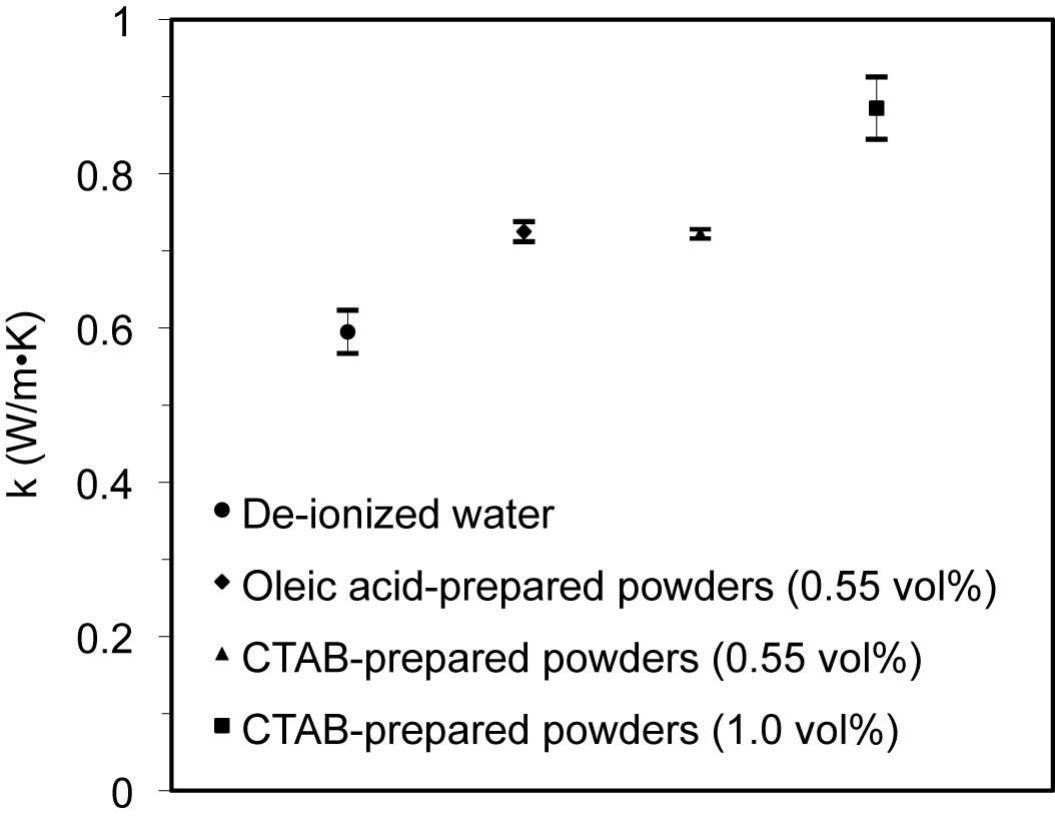
**Dynamic thermal conductivity data**. For de-ionized water and the 0.55 vol.% and 1.0 vol.% nanofluids prepared with the oleic acid- and CTAB-prepared copper powders.

The measured thermal conductivity data for the nanofluids of 0.55 and 1.0 vol.% oleic acid- and CTAB-prepared copper powders is illustrated in Figure 3. The thermal conductivity of the base fluid (i.e., de-ionized water) was measured for comparison with the nanofluids and is marked in the figure. For the 0.55 vol.% copper nanofluids, the thermal conductivity was enhanced from a value of 0.60 W/m·K for pure water to 0.73 and 0.72 W/m·K for the oleic acid- and CTAB-prepared powders, respectively, an enhancement of approximately 22% over water. The 1.0 vol.% nanofluid with CTAB-prepared copper powders exhibited a thermal conductivity of 0.89 W/m·K, which corresponds to a 48% enhancement in thermal conductivity over water. The 1.0 vol.% nanofluid with oleic acid-prepared copper powders settled too rapidly during the measurement, so the thermal conductivity was not obtained.

**Figure 4:**
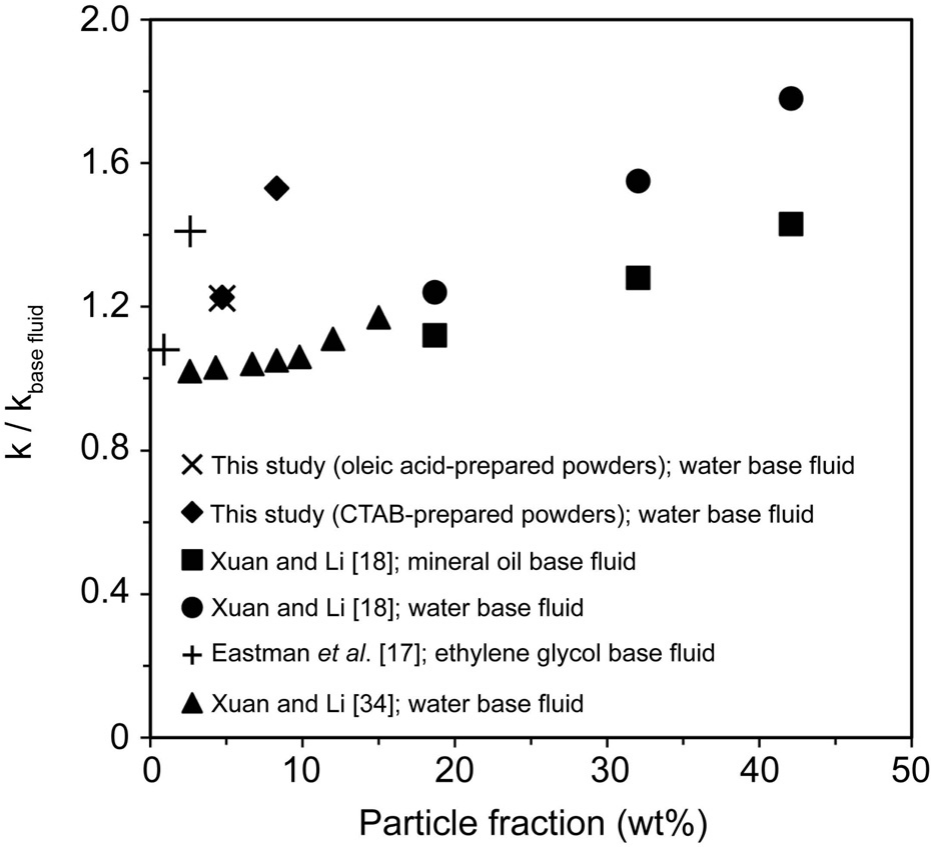
**Comparison of thermal conductivity enhancement for copper-based nanofluids**.

Figure 4 displays a comparison of the thermal conductivity enhancement of the nanofluids produced in this study and three other studies [17,18,34]. This enhancement can be explained using a variety of models which include the microconvection of the nanoparticles due to Brownian motion [35-38], the difference in the thermal conductivity between the dispersed phase and the base fluid [35], liquid layering [39,40], ballistic transport [38,41], and nanoparticle clustering [17,42]. In a complex system, such as a nanofluid, it is likely that the thermal conductivity cannot be explained by just one mechanism. Because the liquid layering of water molecules is rather thin and therefore only applicable to particles <10 nm, this model cannot account for the thermal conductivity enhancement observed in this study [43]. It appears that the most likely cause for the enhancement observed in this study is the combination of the random movements of the particles within the fluid, the ballistic transport of phonons through the particles and the formation of nanoparticle clusters. The microconvection created from the random movements of the nanoparticles in the liquid may not be able to account for a large enhancement, but does play a small role [36]. The Brownian motion of the particles throughout the fluid creates interaction opportunities between the dispersed particles and can increase phonon transport through the fluid [37]. Because copper has a much higher bulk thermal conductivity compared to water, phonons are able to travel through the particles more efficiently. Nanoparticle clusters, or percolating clusters, can form to create lower thermal resistance routes for the propagation of phonons across the fluid. Also, because of the close proximity of the nanoparticles whether due to the Brownian motion, or the formation of percolating structures, or a combination of the two, the ballistic transport of the phonons across the small gaps between particles could account for a significant increase in thermal conductivity [38,41]. We propose that the combination of the formation of the nanoparticle clusters from the Brownian motion of the particles and the ballistic transport of phonons through these clusters and across small gaps between the particles is the main reason for the increase in thermal conductivity observed in this study.

More importantly, we observe that our nanofluid of 1.0 vol.% CTAB-prepared copper powders exhibits comparable or greater enhancement than some nanofluids with much greater particle loadings. For example, the water-based nanofluids prepared by Xuan and Li [18] have particle loadings greater than 30 wt.%, but exhibit almost the same (in fact, slightly lower) thermal conductivity enhancement as our own nanofluids, which have modest particle loadings of 1 vol.% (8.3 wt.%). We attribute this directly to our smaller particle/agglomerate size and possibly our lower viscosity, which plays an important role in dynamic heat transfer applications. An efficient nanofluid involves the greatest thermal conductivity enhancement, with the least amount of particle loading, and therefore, a low viscosity. Thus, we propose that determination of particle/agglomerate size in nanofluids is an important variable that should be determined in order to obtain a complete picture of the characteristics and behavior of nanofluids. An appropriate technique for these measurements is dynamic light scattering.

## Conclusions

Copper nanopowders were successfully synthesized using a chemical precipitation method in the presence of two surfactants, oleic acid and CTAB. This study has explored the importance of particle size distribution analysis in the reporting of nanofluid results. The particle sizes of the powders produced in this study were successfully reduced from approximately 1 μm to 120 and 80 nm for the oleic acid- and CTAB-prepared powders, respectively, upon addition of a dispersant in water. Two particle loadings were used for producing the copper-based nanofluids, 0.55 and 1.0 vol.%. A dynamic thermal conductivity test setup was devised and thermal conductivity measurements were performed on both nanofluids. A thermal conductivity enhancement of 22% over water was observed for the 0.55 vol.% Cu nanofluids. When the particle loading was increased to 1.0 vol.%, the nanofluids of oleic acid-prepared Cu powders settled and clogged the test setup. The nanofluids of CTAB-prepared copper powders remained well dispersed, allowing for a successful thermal conductivity measurement. A maximum increase of 48% was observed for the 1.0 vol.% copper nanofluid from the CTAB-prepared Cu powders. The thermal conductivity enhancement in these latter fluids is directly attributable to the excellent dispersion of the nanoparticles in the fluid.

## Competing interests

The authors declare that they have no competing interests.

## Authors' contributions

MSS carried out the synthesis of the powders, preparation of the nanofluids, and drafted the manuscript. HS completed the thermal conductivity testing. BK designed the thermal conductivity test apparatus and helped to draft the manuscript. YL participated in the design and coordination of the project and helped to draft the manuscript. OAG conceived the study, participated in its design and coordination, and finalized the manuscript.
